# Concomitant confluent and reticulated papillomatosis and acanthosis nigricans in patients with skin of color and need for early interventions for insulin resistance

**DOI:** 10.1097/JW9.0000000000000119

**Published:** 2023-11-03

**Authors:** Ndidi Enwereji, Janelle Mallett

**Affiliations:** a Internal Medicine Department, Henry Ford Hospital, Detroit, Michigan; b Department of Dermatology, University of Connecticut Health Center, Farmington, Connecticut

**Keywords:** acanthosis nigricans, confluent and reticulated, insulin resistance, obesity, papillomatosis, skin of color

**What is known about this subject with regard to women and their families?**

- Diabetes is one of the major cardiovascular disease risk factors.
- Diabetes risk factors are increasing in adolescents.
- Acanthosis Nigricans is considered a cutaneous sign of insulin resistance.

**What is new from this article as messages for women and their families?**

- Better screening for risk factors of insulin resistance in children and adolescents is warranted.
- More studies on assessment and modification of risk factors for insulin resistance are warranted.

## Dear Editors,

### Introduction

Confluent and reticulated papillomatosis (CARP) of Gougerot and Carteaud is an uncommon but unambiguous dermatosis characterized by multiple hyperpigmented reticulated papules and plaques most localized to the upper trunk, neck, and axillae. Histopathology reveals epidermal hyperkeratosis, papillomatosis, and acanthosis.^1^ While the etiology of CARP has been debated in the medical literature,^1,2^ acanthosis nigricans (AN) has been widely established as a cutaneous sign of endocrine and paraneoplastic syndromes. But because CARP can be indistinguishable histologically from AN, the clinical presentation, including response to tetracyclines and anatomic distribution (chest and back), is the clue to diagnosis of CARP. We report on 7 patients with skin of color who presented with both CARP and AN. Dermatologists need to be aware of the increasing association of the 2 skin conditions to help facilitate earlier interventions.

### Summary of cases

**Fig. 1.**
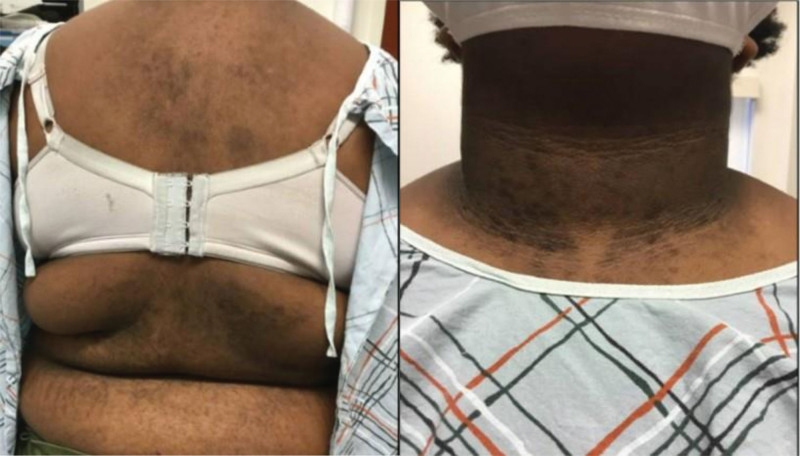
Clinical images of CARP and AN in case #4. (Left) Hyperpigmented and reticulated plaques on the back. (Right) Velvety hyperpigmented papules and plaques on the neck. AN, acanthosis nigricans; CARP, confluent and reticulated papillomatosis.

We present a retrospective case series of 7 patients who were referred to the UCONN Dermatology Outpatient Clinic for evaluation of skin discoloration. The patients’ ages at presentation were between 13 and 29 years; body mass index ranged from 24.5 to 36; and the patients were of Fitzpatrick skin types IV–VI. Physical examination was significant for hyperpigmented and reticulated plaques on the trunk, axillae, and neck (Fig. 1).

All patients were diagnosed with both CARP and AN and were prescribed oral tetracyclines. The trunk (chest and back) eruptions showed only a partial response, some with documented recurrence, while the neck eruptions did not respond to tetracyclines (Table 1).

**Table 1:** Summary of the clinical findings for the 7 patients

Patient	Case 1 (NB)	Case 2 (JR)	Case 3 (LR)	Case 4 (AW)	Case 5 (SW)	Case 6 (AJ)	Case 7 (KE)
Sex, age at diagnosis	F, 29	F, 16	M, 21	F, 14	F, 13	F, 13	F, 25
Dermatological areas affected	FST VI Neck, chest	FST IV Neck, chest	FST V Neck, chest, axilla, thighs	FST VI Neck, chest	FST IV neck, chest, abdomen	FST V chin, neck, chest, elbows	FST V Neck, chest, axilla
BMI	36.02	30.37	34.08	45.6	24.5	31.34	34.86
Obesity class^a^	II-severe	I	I	III-morbid	Nonobese	I	I
Comorbidities	Migraine Asthma Sickle cell anemia	Alopecia	None on file	Moderate obstructive sleep apnea	None on file	None on file	None on file
Medications	Albuterol Ammonium lactate Sumatriptan	Clobetasol 0.05% ointment Tretinoin	None prior to encounter	None prior to encounter	None prior to encounter	None prior to encounter	None prior to encounter
Personal history of diabetes	No	No	No	No	No	No	No
Family history of diabetes	No	No	Yes	Yes	No	Yes	No
Treatment	Minocycline	Minocycline	Minocycline; Ketoconazole 2% shampoo	Minocycline	Doxycycline	Doxycycline	Minocycline; Hydrocortisone 2.5% cream
Response to treatment	Partial, then LTF	Partial with known recurrence	Unknown, LTF	Partial, then LTF	Unknown, LTF	Partial, then LTF	Partial with known recurrence

LTF, lost to follow-up.

a Obesity classification according to body mass index: <18.5 = underweight, 18.6–24.9 = normal weight, 25–29.9 = overweight, 30.0–34.9 = obesity class I, 35.0–39.9 = obesity class II (severe), ≥40.0 = obesity class III (morbid).

### Discussion

Both CARP and AN are difficult to treat. Therapeutic options for AN include topical keratolytic agents (including topical retinoids, ammonium lactate, urea, salicylic acid, and vitamin D analogues), oral treatments (including isotretinoin, acitretin, and metformin), chemical peels, microdermabrasion, laser, and weight loss.^3^

A comprehensive review of 70 studies, including 192 patients with CARP, found that treatment with oral tetracyclines resulted in either complete or partial skin clearance, although recurrence rates also were high.^4^ In contrast, antifungal agents alone showed limited effectiveness.^4^ Other authors recently identified an association between CARP and AN in pediatric patients. Of the 68 patients with CARP, more than half were found to also have AN.^2^ These patients were predominantly Black, overweight or obese; showed signs of insulin resistance and androgen excess, and had a tendency toward treatment resistance. The results of our case study also suggest that CARP in association with AN is possibly more treatment-resistant than CARP alone and is more common in patients with skin of color. Given all the above, and a recent case report showing that weight reduction with bariatric surgery resulted in clearance of CARP,^5^ it is possible that metabolic abnormalities and endocrinopathies play a role in the pathogenesis of CARP. Familial and genetic studies are lacking and need further exploration. Dermatologists can, thus, play a pivotal role in further defining the associations between CARP and AN and help facilitate earlier screening and treatment for any comorbid metabolic disorders, endocrinopathies, and obesity. Nutritional counseling, surgical and pharmacological weight management, and a multidisciplinary approach involving specialists are recommended.

## Conflicts of interest

None.

## Funding

None.

## Study approval

N/A

## Author contributions

All authors significantly contributed to the writing of this article.
